# pH-responsive hydrogel system loaded with curcumin-preconditioned mesenchymal stem cell exosomes for enhanced diabetic wound healing in orthopedic applications

**DOI:** 10.3389/fbioe.2025.1688905

**Published:** 2025-10-20

**Authors:** Senlei Li, Yangyang Li, Yazhou Chen, Jiawen Guo, Qian Zou, Qiuyue Ding

**Affiliations:** ^1^ Department of Orthopedics, Guizhou Provincial People’s Hospital, Guiyang, Guizhou, China; ^2^ Beijing Jishuitan Hospital Guizhou Hospital, Guiyang, Guizhou, China

**Keywords:** pH-responsive, curcumin, anti-bacterial, oxidative stress, immune homeostasis

## Abstract

Wounds, a major complication in orthopedic-related diseases, present significant challenges for orthopedic surgeons due to impaired healing driven by vascular dysfunction, oxidative stress, and chronic inflammation. This study introduces an injectable, self-healing, pH-responsive hydrogel system delivering curcumin-preconditioned mesenchymal stem cell (MSC)-derived exosomes (Cur-Exos) to enhance diabetic wound repair, offering a cell-free therapeutic strategy with applications in orthopedic regenerative medicine. The hydrogel, formed through dynamic cross-linking of amphoteric chitosan and multi-armed polyethylene glycol, exhibits antibacterial properties and degrades selectively in the acidic microenvironment (pH 4.5–6.5) of diabetic wounds. Loaded with Cur-Exos, it enables controlled release of bioactive molecules, leveraging the enhanced antioxidant, anti-inflammatory, and pro-angiogenic properties of exosomes from curcumin-preconditioned MSCs. *In vitro*, Cur-Exo@Gel exhibited excellent biocompatibility, promoted endothelial cell migration, and enhanced angiogenesis, while reducing oxidative stress. It also modulated macrophage polarization by decreasing pro-inflammatory M1 markers (iNOS) via the upregulation of Iκβ-α and reduced p65 phosphorylation, fostering an anti-inflammatory microenvironment. The hydrogel’s inherent antibacterial properties (>50% reduction in *Escherichia coli* and *Staphylococcus aureus*, *p* < 0.05) further mitigate infection risks that are critical in orthopedic wounds. *In vivo*, in a diabetic rat full-thickness skin defect model, Cur-Exo@Gel significantly reduced wound areas, with enhanced epithelial migration and collagen deposition. These findings highlight Cur-Exo@Gel as a promising cell-free therapeutic strategy for accelerating chronic orthopedic wound repair, offering novel insights for regenerative orthopedics.

## 1 Introduction

Chronic wounds associated with orthopedic conditions, particularly in diabetic patients, pose significant challenges in clinical management due to vascular impairment, recurrent infections, and excessive oxidative stress, which collectively impede healing (Xiong et al., 2024; Nowak et al., 2021; Ding et al., 2022a). In orthopedic contexts, such as chronic ulcers or post-surgical complications, improper management can lead to severe outcomes, including extensive tissue damage, sepsis, osteomyelitis, and limb amputation (Powers et al., 2016; Everett and Mathioudakis, 2018). Although various biomaterials have achieved partial success in wound repair, most fail to adapt to the complex diabetic and orthopedic microenvironment, limiting precise tissue regeneration. Consequently, developing systems that respond to the specific microenvironment of orthopedic-related diabetic wounds, enable controlled release of bioactive molecules, mitigate oxidative stress, activate angiogenesis, and restore local blood flow is critical for effective wound repair in orthopedic surgery.

According to the International Diabetes Federation, approximately 9.1 million–26.1 million diabetic patients develop chronic wounds annually, with a 5-year mortality rate reaching 50% (Patel et al., 2019). In orthopedic settings, these wounds incur high treatment costs and remain refractory to conventional therapies (Armstrong et al., 2017). Hyperglycemia and advanced glycation end-products in diabetes elevate local reactive oxygen species (ROS) levels through multiple metabolic pathways (Niu et al., 2012), impairing endothelial cell function, reducing vascular oxygen delivery, and creating a hypoxic microenvironment that hinders orthopedic wound healing (Xu et al., 2020). The physiological pH of wounds is slightly acidic, but in diabetic and infected orthopedic wounds, lactic acid and acetic acid production further lowers the pH to 4.5–6.5 (Rodríguez-Rodríguez et al., 2021; Wu et al., 2022). Hydrogels, characterized by three-dimensional networks of cross-linked hydrophilic polymers, offer excellent biocompatibility and permeability, mimicking the extracellular matrix (Xu et al., 2018). When loaded with therapeutic agents, hydrogels serve as depots for the sustained release of bioactive molecules, enhancing their biological efficacy (Ma et al., 2022; Ding et al., 2022b). Given the variability in the location, size, and depth of orthopedic-related diabetic wounds (Chang and Nguyen, 2021), an injectable, self-healing, pH-responsive hydrogel system capable of targeted bioactive molecule release is essential.

Exosomes, extracellular vesicles (30 nm–200 nm), serve as paracrine mediators of mesenchymal stem cells (MSCs), promoting wound repair (Yu et al., 2020). MSC-derived exosomes carry bioactive molecules, inheriting stem cell regenerative functions while avoiding cellular immune responses, with potent pro-angiogenic capabilities that enable cell-free therapies (Phinney and Pittenger, 2017). However, their limited antioxidant and anti-inflammatory capacities restrict their efficacy in diabetic orthopedic wounds. Previous studies suggest that preconditioning MSCs with physical or chemical stimuli enhances exosome bioactivity (Yu et al., 2020). For instance, Cui et al. demonstrated that exosomes from hypoxia-preconditioned MSCs promote angiogenesis and accelerate diabetic wound healing (Hu et al., 2023). Melatonin-pretreated MSC-derived exosomes promote wound healing by targeting the PTEN/AKT pathway to regulate macrophage polarization (Liu et al., 2020). Curcumin (Cur) is a natural polyphenol with established anti-inflammatory, antioxidant, and antibacterial properties (Yao et al., 2023). Based on emerging evidence that bioactives can enhance the paracrine potential of MSC-derived exosomes, curcumin can be used to precondition bone marrow MSCs, preserving their pro-angiogenic activity while enhancing the antioxidant and anti-inflammatory capacity. Thus, a hydrogel designed for responsive release of curcumin-preconditioned exosomes (Cur-Exos) could leverage the antioxidant, anti-inflammatory, and pro-angiogenic effects to address the challenges of orthopedic wound repair.

In this study, we developed a pH-responsive, injectable, and self-healing hydrogel tailored for the weakly acidic microenvironment (pH 4.5–6.5) of diabetic orthopedic wounds. The hydrogel is formed through Schiff base cross-linking between amphoteric carboxymethyl chitosan (CMC) and four-armed polyethylene glycol benzaldehyde (PEGBA). CMC’s inherent antibacterial properties protect against external pathogens and biofilm formation, which is critical for preventing infections in orthopedic wounds (Confederat et al., 2021). The reversible Schiff base bonds confer pH- and shear-responsiveness, enabling specific degradation and controlled release of Cur-Exos in the acidic wound environment (Zhan et al., 2020). Schiff base cross-linking was widely utilized in wound healing applications due to its responsiveness to acidic microenvironments, which is a hallmark of diabetic and chronic wounds. This design ensures not only injectability and self-healing properties but also promotes structural adaptation within the physiological wound milieu (Zhang et al., 2025; Dai et al., 2024).

This system activates antioxidant defenses, mitigates excessive oxidative stress in endothelial cells, promotes endothelial cell migration, and enhances angiogenesis. Additionally, it modulates macrophage polarization toward the M2 phenotype, reducing pro-inflammatory M1 activity. *In vivo*, Cur-Exo@Gel significantly reduced wound areas in a diabetic rat full-thickness skin defect model with enhanced epithelial migration and collagen deposition. These findings position Cur-Exo@Gel as a promising cell-free therapeutic strategy for accelerating the repair of chronic orthopedic wounds, offering novel insights for clinical translation in regenerative orthopedics.

## 2 Methods

### 2.1 Synthesis of benzaldehyde-terminated four-arm PEG

Four-arm polyethylene glycol (PEG, 2.00 g, 0.2 mmol), 4-formylbenzoic acid (0.24 g, 1.6 mmol), and 4-dimethylaminopyridine (DMAP, 0.049 g, 0.4 mmol) were dissolved in 100 mL of anhydrous tetrahydrofuran (THF). N,N′-dicyclohexylcarbodiimide (DCC, 0.41 g, 2 mmol) was added, and the mixture was stirred magnetically under a nitrogen atmosphere for 72 h. The product was purified by dissolving in THF, washing three times with cold absolute ethanol, and drying in a vacuum oven at 25 °C for 72 h. The chemical structure of PEG-BA was characterized using Fourier-transform infrared spectroscopy (FT-IR) and ^1^H nuclear magnetic resonance (^1^H NMR, 400 MHz, D_2_O).

### 2.2 Synthesis of carboxymethyl chitosan

Carboxymethyl chitosan (CMC) was synthesized following a previously reported method (Xiong et al., 2024). In brief, chitosan (10 g) was dispersed in a 50 wt% NaOH aqueous solution and frozen at −20 C for 12 h. The mixture was transferred to a flask containing isopropanol (100 mL), followed by the gradual addition of sodium chloroacetate (35 g). The reaction proceeded with stirring at 22 C for 2 h and then at 60 C for an additional 2 h. The reaction was quenched with 70% ethanol, and the precipitate was washed sequentially with 70% ethanol and absolute ethanol and then dried at 60 C. The resulting CMC was characterized by FT-IR and ^1^H NMR (400 MHz, D_2_O).

### 2.3 Preparation of CMC/PEG-BA hydrogels

A 2% (w/v) CMC solution was prepared by dissolving 200 mg of CMC in 10 mL of ultrapure water at room temperature. Similarly, a 2.5% (w/v) PEG-BA solution was obtained by dissolving 250 mg of PEG-BA in 10 mL of ultrapure water. To form the CMC/PEG-BA hydrogel, equal volumes of the CMC and PEG-BA solutions were mixed, vortexed for 30 s, and incubated at 25 C to allow gelation.

### 2.4 Extraction and identification of BMSCs and BMSC Exos

Bone marrow stromal cells (BMSCs) were isolated and cultured following the instructions mentioned in a previous study (Hu et al., 2021). Then, it was confirmed via flow cytometry (FCM) with positive markers CD9 and CD73 and the negative marker CD45. BMSCs were preconditioned with 10 µM Cur for 24 h with exosome-free fetal bovine serum. The supernatant medium was harvested for centrifugation at 15,000 ×g to remove dead cells for 35 min. Then, 100,000 ×g centrifugation was carried out for 75 min twice. Finally, the collected Exo was resuspended with phosphate-buffered saline (PBS) before being kept at −80 C for further experiments. The ultrastructure and shape of Cur-Exos and NC-Exos were observed via transmission electron microscopy (TEM, FEI Talos F200S Waltham, United States). Meanwhile, the particle size of exosomes was evaluated by nanoparticle tracking analysis (NTA, Munich, Germany ZetaView PMX 110, Particle Metrix). CD63, CD9, and calnexin were detected by Western blotting.

### 2.5 Cell survival and migration assay *in vitro*

To assess the impact of exosomes on cell migration, human umbilical vein endothelial cells (HUVECs) were seeded in 24-well plates for 24 h. Cells were then serum-starved overnight to synchronize their growth state. A linear micro-injury was created using a 200 μL pipette tip, followed by washing with PBS to remove debris. HUVECs were treated with exosomes at a concentration of 20 μg/mL, and wound closure was monitored and imaged over 24 h. Wound areas were quantified using ImageJ software to calculate the percentage of wound closure and migration rate. To evaluate the effects of exosomes on cell survival, HUVECs were exposed to treatments from different groups for 24 h–48 h. Cell viability was assessed using a calcein AM/propidium iodide (PI) live–dead assay, which determined the proportion of viable cells.

### 2.6 Reactive oxygen species assay and tube formation assay

The cellular ROS scavenging activity was measured using the reactive oxygen species assay kit (DCFH-DA). In brief, HUVECs cultured in 6-well plates were divided into five groups: control, T-BHP, PEG-CMC, NC-Exo@, and Cur-Exo@. Except for the control group, all other groups were exposed to 75 μM t-BHP for 6 h. Cells in each group were then stained with 10 μM DCFH-DA at 37 C for 30 min in the dark. Intracellular ROS levels were subsequently observed using a fluorescence microscope or flow cytometer. To assess the capillary-like structure formation capacity in the different treatment groups, a tube formation assay was performed. In brief, Ceturegel™ Matrix LDEV Free (YEASEN, Shanghai, China 40183ES08) was fully melted at 4 C overnight. Then, 50 μL of Ceturegel™ Matrix was added to a pre-cooled 96-well plate, which was then placed in a culture incubator for 30 min for future use. HUVECs from the different pretreated groups were seeded at a density of 2 × 10^5^ cells per well on the 96-well plate and incubated for 4 h. The tube formation ability was verified using an inverted light microscope and analyzed via ImageJ.

### 2.7 Flow cytometry and immunofluorescence staining

Flow cytometry was performed to detect macrophage polarization. RAW264.7 cells were cultured in different groups and then resuspended at a density of 10^6^ cells/100 μL before being used. In brief, before incubation with surface antigen CD206 for 30 min, the suspensions were treated with Fc blocker at 4 C for 10 min. For intracellular antigen staining, the cells were incubated with primary antibodies CD11C and CD86 (Abcam). They were then washed two times with cell-staining buffer according to the manufacturer’s recommendations. Finally, the cells were resuspended and analyzed by flow cytometry (BD). Each analysis was performed with data from at least three independent experiments. The data were analyzed using FlowJo V10.0 (Tree Star, Ashland, OR, United States).

RAW264.7 cells were cultured in different groups for 3 days, followed by fixation with 4% paraformaldehyde for 25 min. The cells were then permeabilized with 0.2% Triton X-100 and blocked with 5% bovine serum albumin for 30 min at room temperature. After incubation with primary antibodies CD206 (Abcam), CD163 (Abcam), and iNOS (Abcam) overnight at 4 C, the cells were treated with secondary antibodies (Proteintech, Wuhan, China) for 30 min in the dark. Nuclei were stained with 4′,6-diamidino-2-phenylindole (DAPI, Beyotime Biotechnology, China) for 5 min. Finally, immunofluorescence images were captured using a fluorescence microscope (Olympus, IX73), and the fluorescence intensity was quantified using ImageJ software (National Institute of Health, United States).

### 2.8 Western blotting

RAW264.7 cells or exosomes were harvested, washed two twice, and then lysed with RIPA lysis buffer. The BCA method was applied to detect the protein concentration. After being separated by SDS-PAGE gel electrophoresis, the protein was transferred to a PVDF membrane. Before incubation with primary antibodies such as Ikβ-α, p65, p-p65, CD63, CD9, calnexin, and GAPDH at 4 °C overnight, the membrane was blocked with 5% BSA for 1 h at room temperature. Finally, target protein expression was visualized using the enhanced chemiluminescence assay.

### 2.9 Wound healing experiments in an animal model

All SD rats (male, 300 g ± 30 g) were provided by the Guizhou University of Traditional Chinese Medicine (Guiyang, China). A rat model of diabetes was established as in prior reports (Ding et al., 2022a). In brief, diabetes in rats was induced by intravenous injection of streptozotocin (STZ). After the blood glucose levels exceeded 16.7 mM for four consecutive days, the diabetic model was considered successful. The diabetic SD rats were then randomized into the control, PEG-CMC, NC-Exo@, and Cur-Exo@ groups. Rats were anesthetized using a small animal anesthesia machine (RWD Life Science, Shenzhen, China R500) delivering isoflurane (Salisbury, United States IsoFlo, Zoetis). Rats were anesthetized with 3%–5% isoflurane in 100% oxygen (flow rate: 1 L/min–2 L/min) until loss of consciousness, followed by maintenance at 1.5%–2.5% isoflurane during the creation of a 15 mm × 15 mm square full-thickness wound. Wound healing was recorded using a digital camera on days 0, 5, and 10 following wound establishment. On day 10, rats of each group were sacrificed. Euthanasia was performed using carbon dioxide (CO_2_) inhalation in a dedicated chamber, with a gradual fill rate of 30%–70% chamber volume per minute to induce unconsciousness, followed by sustained exposure until cessation of respiration and cardiac activity was confirmed. Cervical dislocation was performed as a secondary method to ensure death. Wound site tissue samples were harvested for hematoxylin and eosin (H&E) staining, Masson’s trichrome staining, and immunohistochemical (IHC) staining. The change in wound area was analyzed using ImageJ.

### 2.10 Histological analysis and immunohistochemical staining

The wound tissues were fixed in 4% polyformaldehyde and embedded in paraffin. Then, the tissue was cut into 5 μm-thick sections for histological analysis. H&E and Masson’s trichrome staining were applied to observe the regeneration ability and collagen formation in different treatment methods. IHC staining was performed according to the standard protocols. After dewaxing to remove the paraffin and hydration to adapt to the water-based media, the sections were washed in PBS and quenched by immersion in 3% (v/v) hydrogen peroxide for 5 min. Sections were incubated with the following primary antibodies: anti-iNOS (BA0131, Boster Biological Technology, China) and anti-CD206 (Boster Biological Technology, China). Three animals per group were analyzed for IHC staining. For each sample, we randomly selected at least five fields for analysis.

### 2.11 Statistical analysis

Data are presented as the means ± SD from three or more analyses. GraphPad Prism was used to compare data using one-way ANOVAs with Tukey’s test. *p* < 0.05 was the significance threshold (NS, not significant; *p < 0.05, **p < 0.01, and ***p < 0.001).

## 3 Results and discussion

### 3.1 Preparation and characterization of BMSCs and Cur-Exos

**FIGURE 1:**
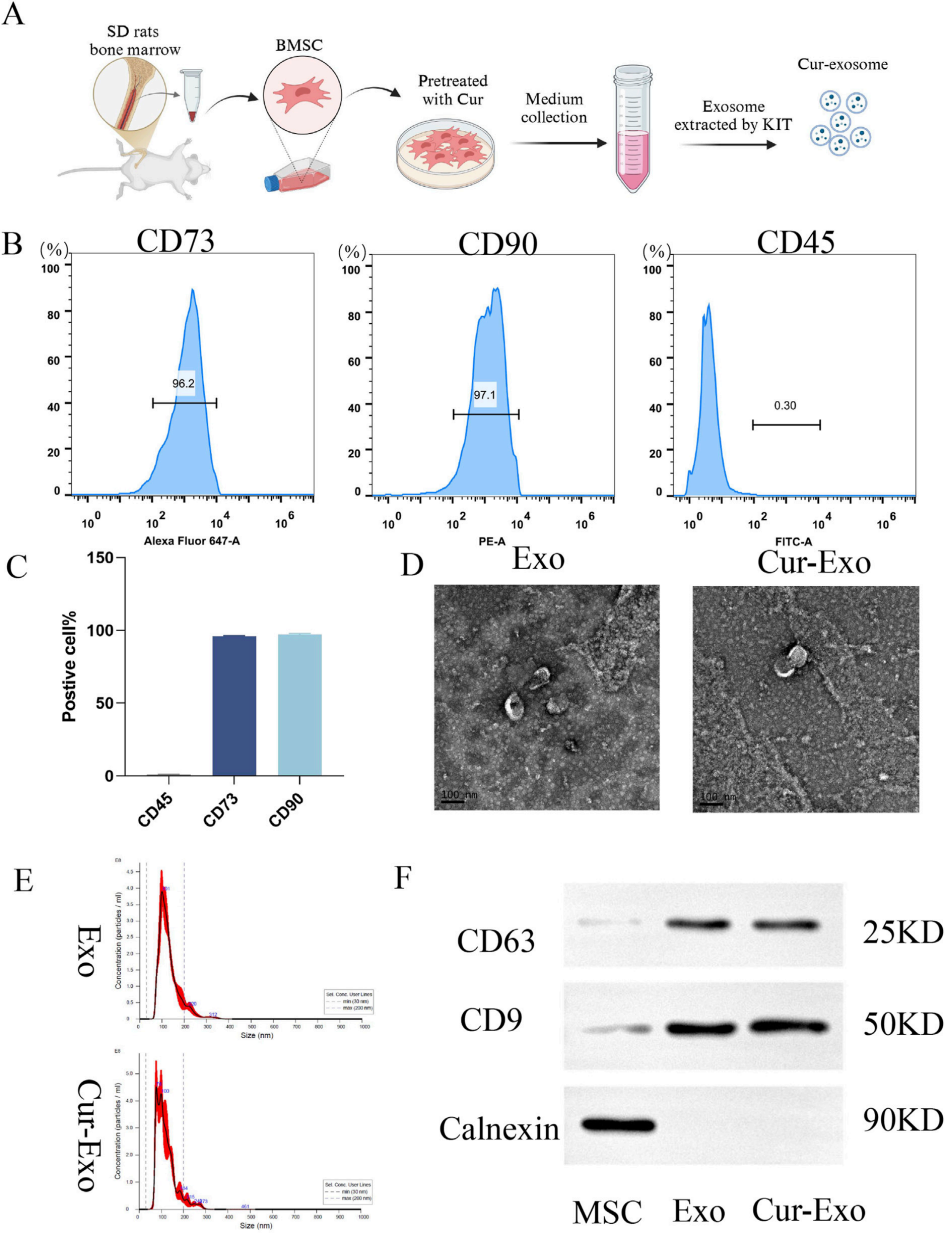
Extraction and characterization of exosomes. **(A)** Extraction process of BMSC-derived exosomes. **(B,C)** Flow cytometry identification of surface markers of BMSCs. **(D)** Transmission electron microscopy images of NC-Exo/Cur-Exo. **(E)** Nanoparticle tracking analysis of the particle size distribution of NC-Exo/Cur-Exo. **(F)** Western blot analysis of markers of BMSCs, NC-Exo, and Cur-Exo.

First, the isolated and cultured BMSCs were identified using optical images and flow-cytometric analysis. As shown in Figure 1A, the BMSCs were obtained from the SD rat. The FCM data confirmed that BMSCs highly expressed the positive markers CD90 and CD73 but expressed very low levels of the negative marker CD45. The expression rates were 97.1%, 96.2%, and 0.30%, respectively (Figure 1B). The above results demonstrated that BMSCs were successfully extracted and exhibited MSC properties, consistent with previous studies. The process of Cur-exosome preparation is shown in Figure 1A. BMSCs were incubated with curcumin to collect the supernatant. Exosomes were then isolated from the supernatant of BMSCs with or without Cur by ultracentrifugation and characterized via NTA, TEM, and Western blotting. As illustrated in Figures 1C–E, both the morphology of Exo and Cur-Exo had typical exosomal structures with significant lipid bilayer membrane vesicles. Their diameter was approximately 120 nm, with no significant difference between them. NTA data showed that the sizes of the Exo and Cur-Exo ranged from 30 to 300 nm, with no significant differences between them. The exosomal surface markers such as CD9 and CD63 were detected by Western blot analysis in the Exo and Cur-Exo groups, but the negative marker calnexin was not expressed in them (Figure 1F). Together, these results confirmed that the isolated Cur-Exos meet the standard criteria for BMSC-derived exosomes.

### 3.2 Synthesis, injectable, and self-healing performances of hydrogels

**FIGURE 2:**
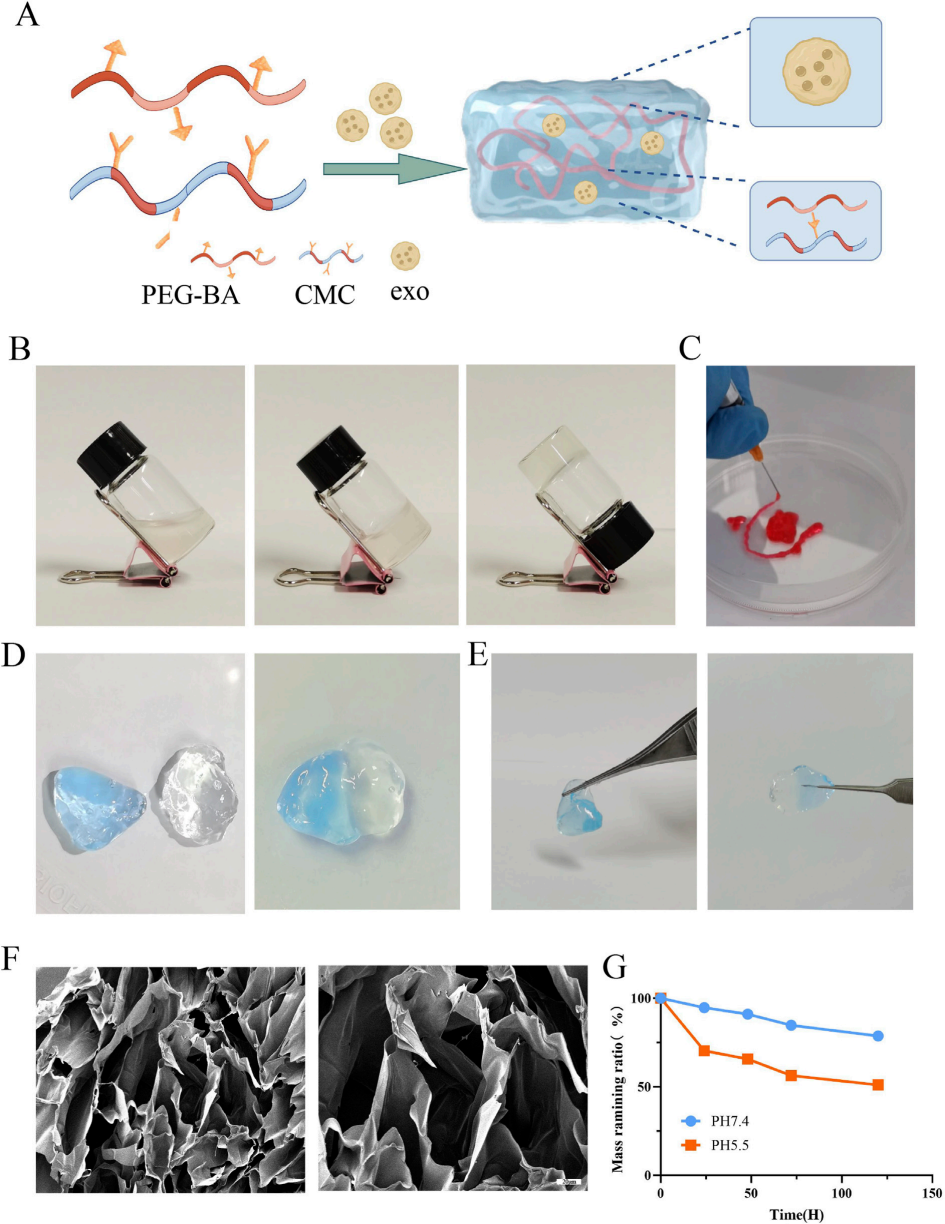
Preparation and characterization of Cur-Exo@Gel. **(A)** Schematic of hydrogel synthesis. **(B)** Gelling process of the hydrogel. **(C)** Injectable ability of the hydrogel. **(D, E)** Self-healing mechanism. **(F)** SEM analysis of the PEG-CMC hydrogel. **(G)** Degradation of the PEG-CMC hydrogel on different pH PBS solutions.

The process of synthesis of PEG-CMC hydrogels, NC-Exo@, and Cur-Exo@ is shown in Figure 2A. The PEG-CMC hydrogel was based on CMC and 4-arm PEG-BA at room temperature. After being mixed with CMC, the 4-arm PEG-BA solution can easily change into a stable hydrogel in a few seconds (Figure 2B). As shown in Figure 2C, the hydrogel can be easily injected using a syringe needle with an inner diameter of 0.5 mm and was dyed with Rhodamine B. The self-adhesive properties are shown in Figure 2D, E. One part was dyed blue with methylene blue, while the other part was left undyed. After being attached for 10 min, the two parts of the hydrogel bound together and could be lifted with tweezers in any direction without falling apart. Scanning electron microscopy analysis revealed that the hydrogel exhibits a highly organized porous architecture, facilitating efficient channels for nutrient and gas exchange (Figure 2F). The pH-responsive degradation behavior of the hydrogel was assessed, and the results are presented in Figure 2G. The hydrogel exhibited significantly faster degradation at pH 5.5 than at pH 7.4, highlighting its sensitivity to acidic environments. In general, the injectability and self-healing ability of the hydrogel indicate that it can adapt to diabetic wounds of different shapes. As an exosome carrier, the pH-sensitive property of the hydrogel is beneficial for exosome release in a local diabetic environment. All these attributes suggest that the hydrogel is a promising responsive carrier for improving diabetic wound healing.

### 3.3 *In vitro* release of exosome, biocompatibility, and antibacterial activity of hydrogels

**FIGURE 3:**
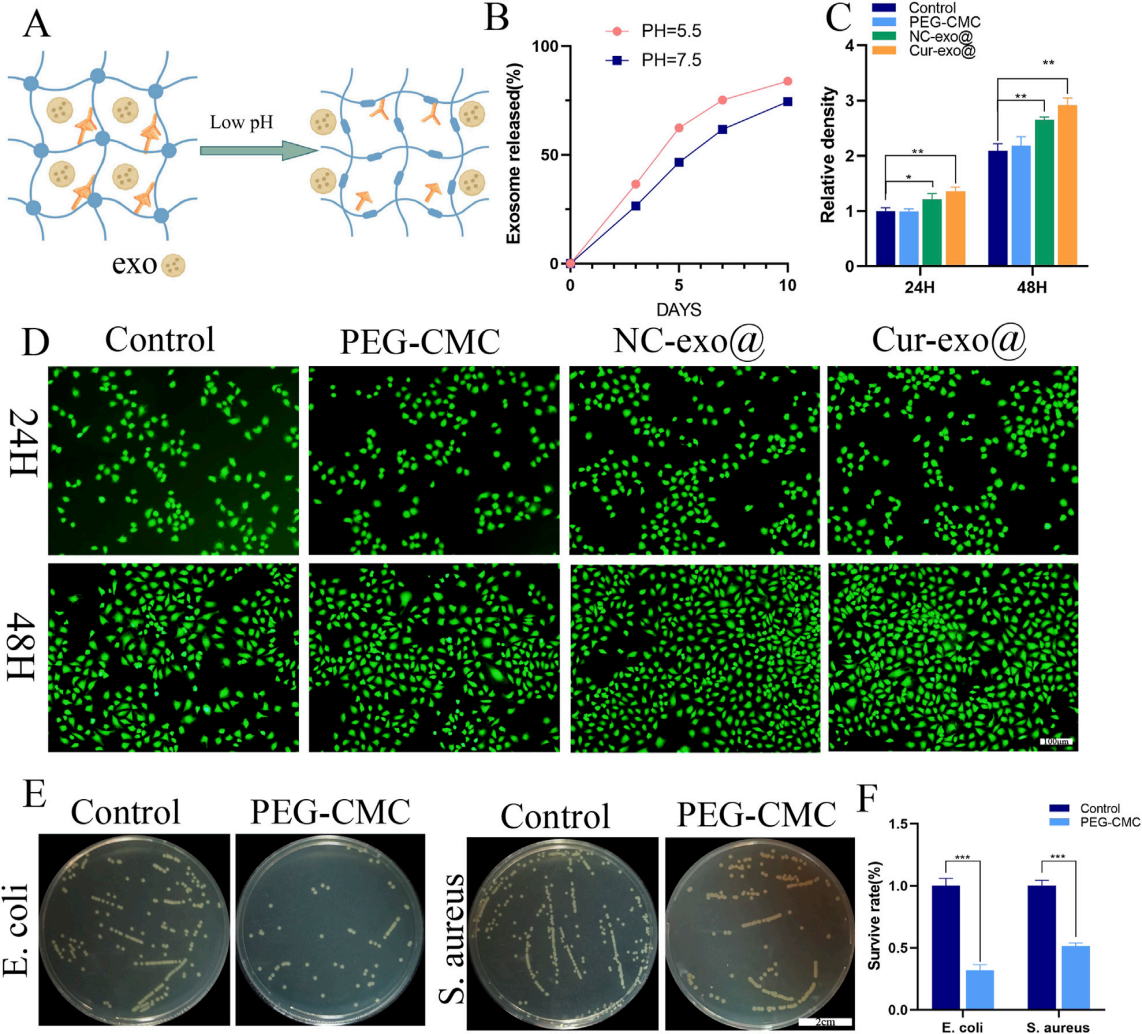
Characterization and biological properties of the hydrogel. **(A)** pH-responsive process. **(B)** pH-responsive release of loaded exosomes in the hydrogel. **(C)** Fluorescence intensity and **(D)** live staining of HUVECs for different hydrogel groups. **(E, F)** Antibacterial activity of the hydrogel against *Staphylococcus aureus* and *Escherichia coli*.

Chronic wounds associated with diabetes and other orthopedic conditions are highly susceptible to bacterial infections, resulting in persistent inflammation and a weakly acidic cellular microenvironment (Chen et al., 2024). The exosome-loaded hydrogel developed in this study is designed to release its bioactive cargo in response to such acidic conditions, as illustrated in Figure 3A, which models the acidic wound environment at pH 5.5. As shown in Figure 3B, the hydrogel exhibits significantly faster exosome release at pH 5.5 than at a neutral pH of 7.4, indicating its pH-responsive behavior and the ability to rapidly deliver therapeutic exosomes in the acidic microenvironment of chronic wounds, thereby enhancing biological efficacy. The release profile is consistent with established pH-responsive hydrogels, as reported in previous studies. This biphasic release kinetics is strategically aligned with the temporal requirements of wound healing, facilitating rapid anti-inflammatory action followed by prolonged therapeutic activity. Since the hydrogel directly contacts cells, its excellent biocompatibility is critical for promoting tissue regeneration. Biocompatibility implies that the material exhibits minimal toxicity to normal cells. In this study, the biocompatibility of the hydrogel was evaluated *in vitro* using a live/dead staining assay with HUVECs. As shown in Figures 3C, D, HUVECs cultured in hydrogel extracts for 24 h and 48 h exhibited complete cell spreading across all groups. The fluorescence intensity of the hydrogel group was comparable to or exceeded that of the control group, while the exosome-loaded hydrogel group demonstrated significantly higher fluorescence intensity relative to the control, with all differences achieving statistical significance (*p* < 0.05). This result demonstrates that the PEG-CMC hydrogel exhibits excellent biocompatibility and the exosome-loaded hydrogel can promote HUVEC proliferation.

Beyond their superior pH-responsive properties and biocompatibility, wound dressing materials with intrinsic antibacterial activity are more advantageous for enhancing wound healing in orthopedic applications. The antibacterial activity of the PEG-CMC hydrogel dressing was evaluated using *Escherichia coli* (a Gram-negative bacterium) and *Staphylococcus aureus* (a Gram-positive bacterium). Compared to that in the control group, the number of plate colonies in the PEG-CMC hydrogel group was reduced by more than 50% for both *E. coli* and *S. aureus*, demonstrating that the PEG-CMC hydrogel has inherent antibacterial properties. The antibacterial efficacy of the PEG-CMC hydrogel is attributed to the Schiff base active bonds and protonated amino groups, which bind to negatively charged bacterial surfaces, thus disrupting their membranes and causing bacterial death. In summary, the PEG-CMC hydrogel exhibits pH-responsive properties, enabling exosome release in the acidic microenvironment of orthopedic wounds. Additionally, its excellent biocompatibility and potent antibacterial activity make it an ideal wound dressing for orthopedic applications.

### 3.4 Antioxidant effects to promote angiogenesis of Cur-Exo@ gels

**FIGURE 4:**
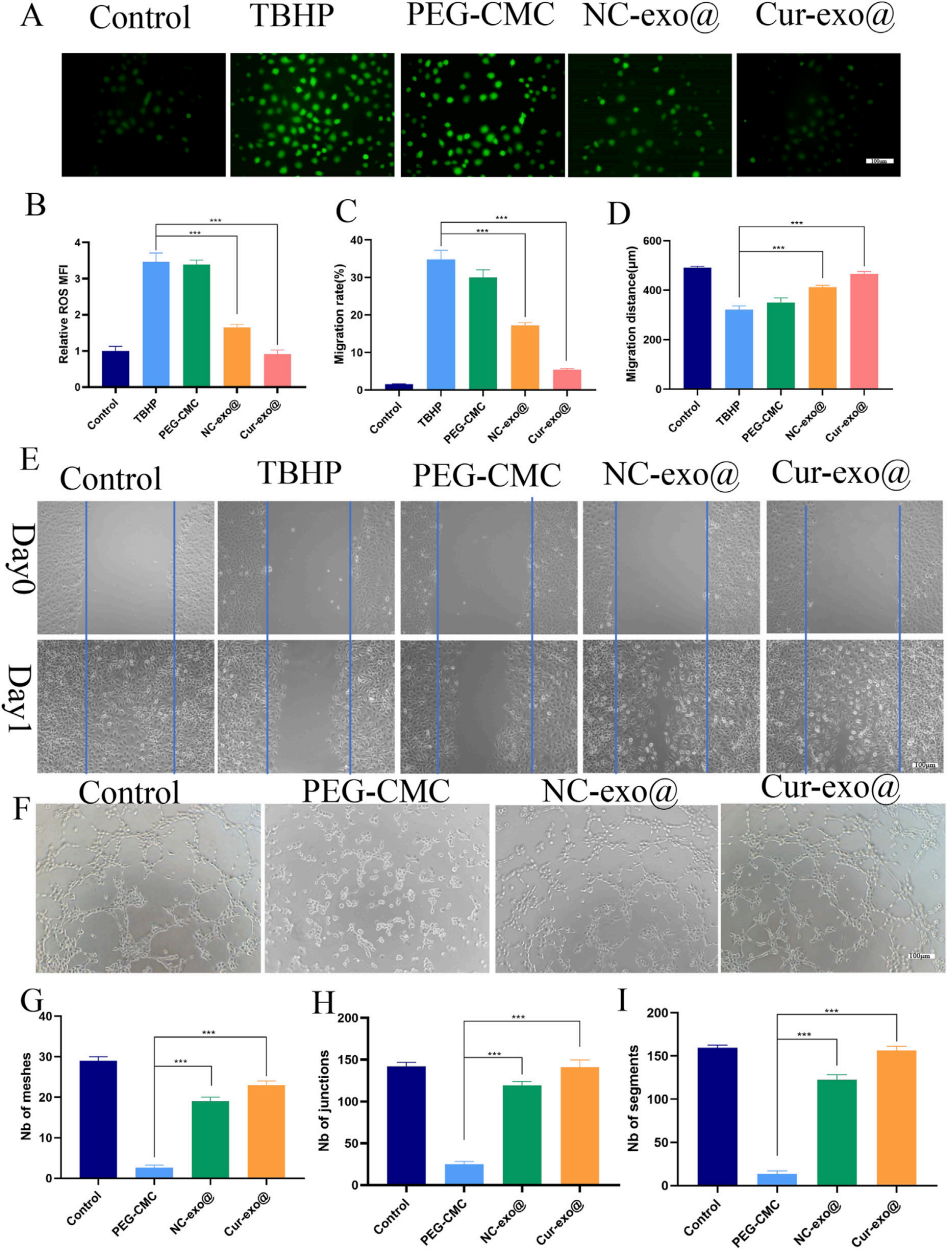
Antioxidant, cell migration, and angiogenic capacity of the hydrogel. **(A)** Representative ROS staining of HUVECs. **(B)** Quantitative analysis of intracellular ROS levels in HUVECs. **(C-E)** Cell scratch assay and quantitative analysis of HUVECs treated with the hydrogel. **(F-I)** Tube formation assay and quantitative analysis of HUVECs treated with the hydrogel.

Chronic wounds in orthopedic contexts are frequently complicated by a hypoxic and hyperglycemic microenvironment, resulting in the excessive production of ROS (Weng et al., 2025). Elevated ROS levels exacerbate tissue inflammation, induce cellular necrosis, and impair the tissue repair process. Consequently, attenuating excessive oxidative stress in the wound microenvironment is essential for facilitating effective wound repair in orthopedic applications. Therefore, we investigated the antioxidant effects of the hydrogel in HUVECs loaded with DCFH-DA. The HUVECs were pre-treated with different hydrogel extracts for 12 h and then exposed to 75 μm TBHP: tert-Butyl hydroperoxide to simulate oxidative stress. As shown in Figures 4A, B, cells loaded with the Cur-Exo hydrogel group have lower fluorescence intensity than the TBHP-treated groups. The mean fluorescence intensity in the NC-Exo hydrogel group was less than half of that in the TBHP group. These findings demonstrate that exosomes derived from BMSCs and Cur-Exo possess significant antioxidant properties. Notably, the Cur-Exo group exhibited the highest antioxidant efficacy, highlighting its potential for mitigating oxidative stress in orthopedic wound-healing applications. Although previous antioxidant systems (e.g., Prussian blue nanoparticles, MXenes) demonstrate potent ROS scavenging, they often lack biological functionality (Xu et al., 2022; Li et al., 2022). In contrast, Cur-Exo@Gel combines ROS clearance with pro-healing signals from exosomes, offering a multifunctional therapeutic strategy.

Angiogenesis represents a pivotal mechanism in wound healing, particularly in orthopedic contexts, where cell migration constitutes a crucial step in the formation of new blood vessels. Subsequently, we further investigated the angiogenesis capacity of HUVECs treated with different hydrogel groups. After co-culturing the cells with hydrogel eluate for 24 h, the scratch assay was carried out to evaluate the effect of hydrogel on HUVEC migration (Figures 4C–E). The findings reveal that the Cur-Exo hydrogel group achieved the most significant wound closure, with only 5% of the wound area remaining, while it was 13% for the NC-Exo group (Figure 4C). Consistent with wound area measurements, the migration distance of the Cur-Exo hydrogel group was the longest (Figure 4D). The tube formation assay was performed to detect the angiogenic ability of different hydrogel groups. Then, pretreated HUVECs were harvested and seeded on the surface of matrix materials for 4 h. Compared with the PEG-CMC group, the number of meshes, junctions, and segments in the Cur-Exo@gel group was higher, approaching the levels observed in the control group (Figures 4F–I). The results revealed that curcumin-pretreated BMSC exosomes have antioxidant effects and promote angiogenesis ability, indicating that their functional properties were enhanced by curcumin. In summary, the Cur-Exo@Gel not only exerts antioxidant effects but also promotes endothelial cell migration, enhances *in vitro* angiogenesis, improves local blood supply, and strengthens the capacity to facilitate rapid repair of orthopedic-related wounds.

### 3.5 Polarization of macrophages on the Cur-Exo@Gel *in vitro*

Chronic wounds are exposed to excessive inflammatory stimuli, resulting in sustained pro-inflammatory (M1) polarization of resident macrophages. Modulating macrophage polarization to reshape the inflammatory microenvironment is pivotal for biomaterial-mediated wound healing. In this study, RAW264.7 macrophages were treated with lipopolysaccharide (LPS) to induce an inflammatory response, mimicking the chronic wound microenvironment. Under the inflammatory conditions, macrophages predominantly maintain an M1 phenotype, contributing to persistent inflammation.

**FIGURE 5:**
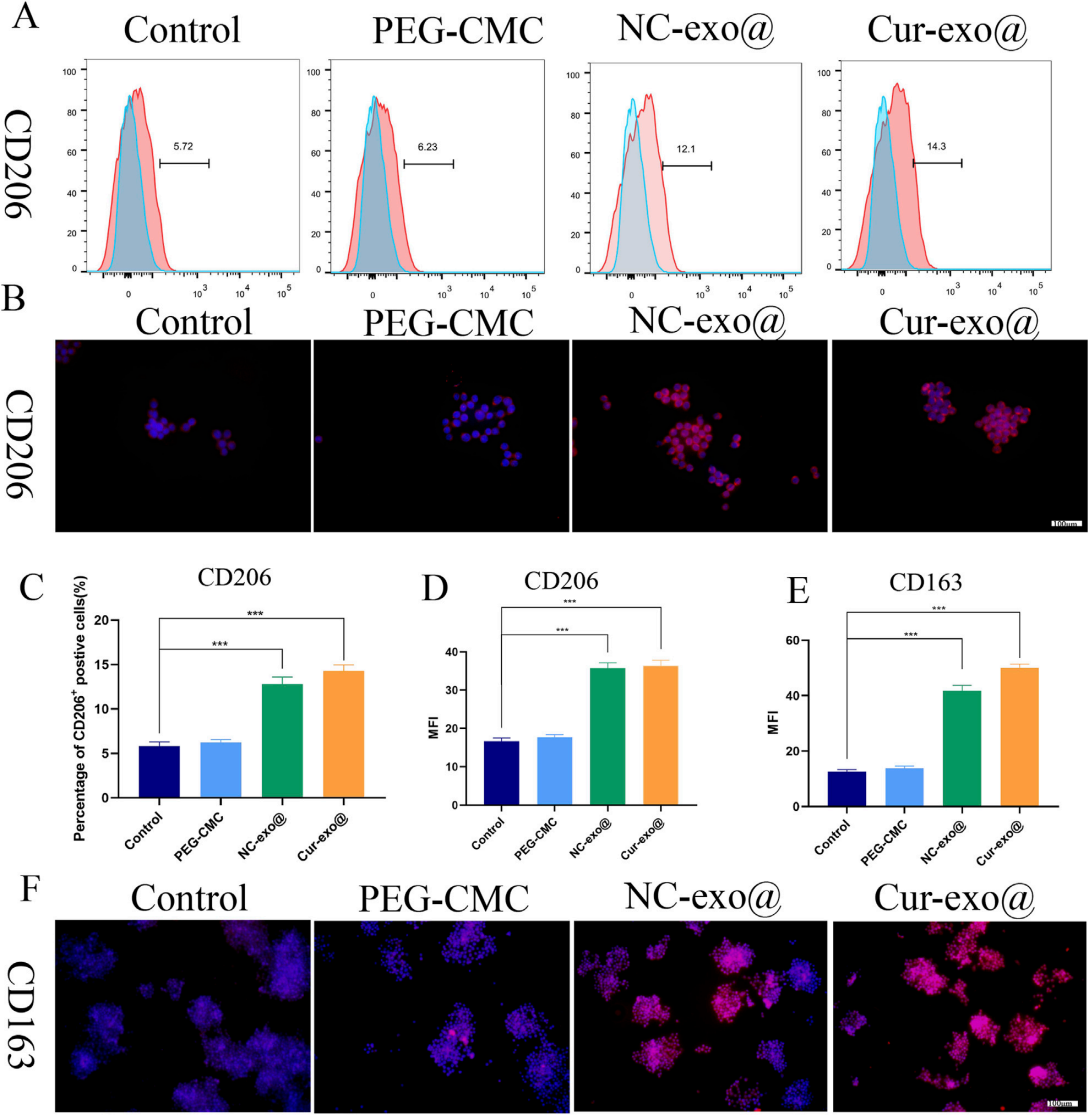
Hydrogel-induced macrophage M2 polarization *in vitro*. **(A)** Flow cytometry analysis of the macrophage surface marker CD206. **(B)** Immunofluorescence of CD206 in macrophages treated with different hydrogels. **(C)** Quantitative analysis of flow cytometry in CD206. **(D)** Mean fluorescence intensity of CD206 in macrophages treated with different hydrogels. **(E, F)** Quantitative analysis and immunofluorescence of CD163 in macrophages treated with different hydrogels.

Reducing the proportion of M1 macrophages while increasing M2 macrophage populations is essential for alleviating local inflammation and promoting tissue regeneration. CD206 and CD163 are the markers of M2 macrophages, while CD11C, CD86, and iNOS are the markers of M1 macrophages. To further investigate the effects of Cur-Exo@Gel on macrophage polarization, flow cytometry and immunofluorescence analyses were conducted. As depicted in Figures 5A, C, the Cur-Exo@Gel group exhibited a significantly higher proportion of CD206-positive RAW264.7 macrophages (14.3%) than the LPS-treated group (5.72%). The immunofluorescence analysis of CD206 expression revealed that exosome-loaded hydrogel groups displayed significantly higher fluorescence intensity than the control group (*p* < 0.05) (Figures Fig 5B, D). However, no statistically significant differences were observed among the various exosome-containing groups. The exosome-loaded hydrogel group exhibited elevated fluorescence intensity of CD163-positive macrophages compared to the control group (*p* < 0.05). The results demonstrate that the exosome-loaded hydrogel promotes M2 macrophage polarization. However, the Cur-Exo hydrogel does not show a strong effect on M2 polarization.

**FIGURE 6:**
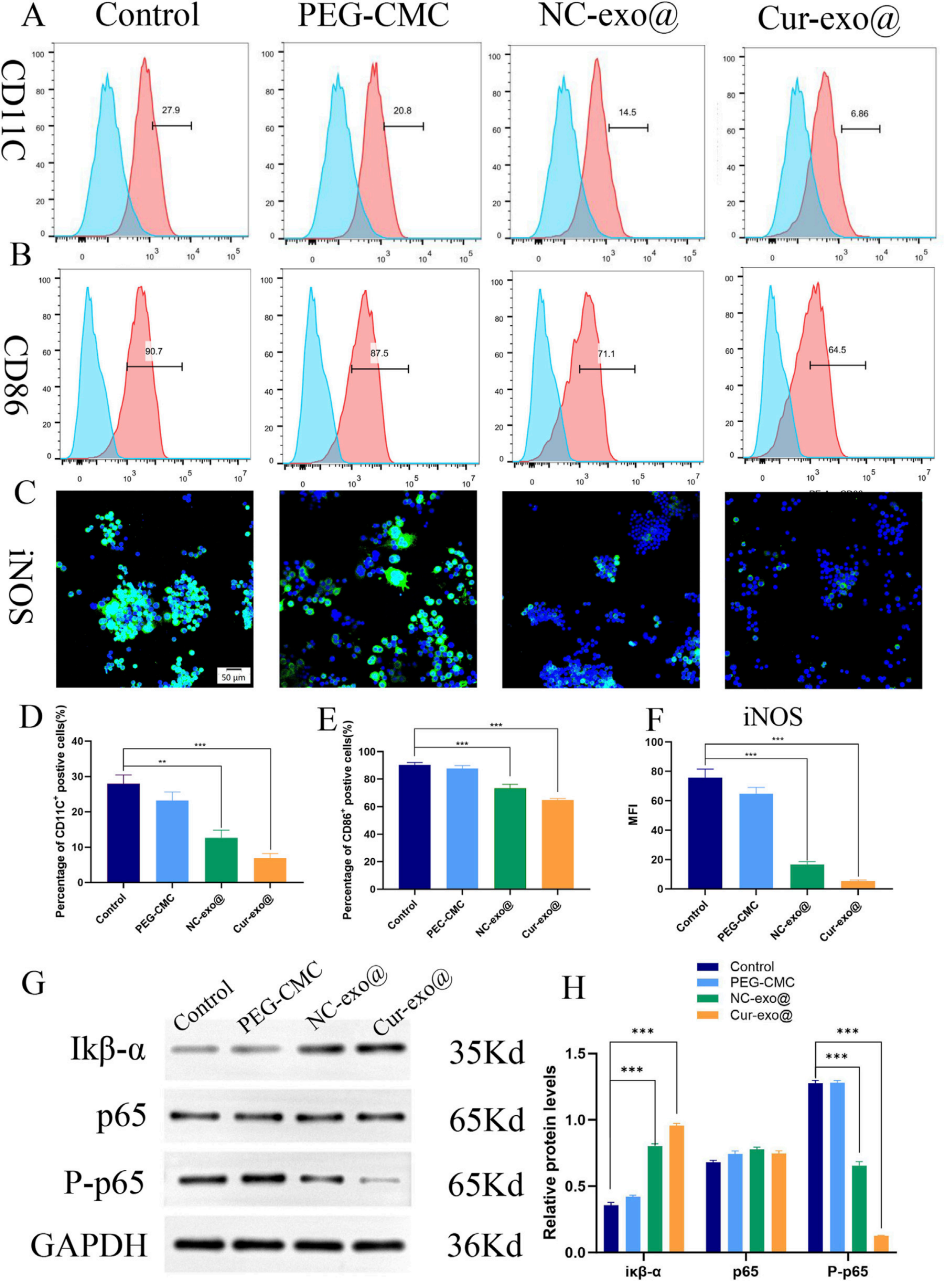
Immunomodulatory properties pathway of the hydrogel. **(A, B)** Flow cytometry analysis of the macrophage surface markers CD11C and CD86. **(C)** Representative immunofluorescence images of iNOS in RAW264.7 cells. **(D, E)** Quantitative analysis of flow cytometry in CD11C and CD86. **(F)** Quantitative analysis of the mean fluorescence intensity of iNOS. **(G)** Western blot of IκB-α, p65, and p-p65. **(H)** Quantitative analysis of the relative expression of IκB-α, p65, and p-p65 in different hydrogels.

As shown in Figures 6A, D, the proportion of CD86^+^ macrophages (M1-type) decreased to 6.86% in the Cur-Exo@Gel group compared to that in the control group (27.9%). Meanwhile, the percentage of CD86^+^ macrophages in the Cur-Exo@Gel group (64.5%) was lower than that in the control group (90.7%) (Figures 6B, E). The expression of the classical M1 macrophage marker iNOS was significantly reduced after treatment with Cur-Exo@Gel (Figures 6C, F). Therefore, the findings indicate that Cur-Exo@Gel significantly reduces the proportion of pro-inflammatory M1 macrophages, as evidenced by flow cytometry and immunofluorescence analyses. The modest upregulation of M2 markers may be attributed to the specific curcumin concentration used (10 μm), which preferentially suppresses M1 polarization. Previous studies have established that curcumin exerts anti-inflammatory effects by inhibiting NF-κB p65 phosphorylation (Peng et al., 2021), primarily by stabilizing IκB-α and suppressing IκB kinase (IKK) activity. To elucidate the underlying mechanism of Cur-Exo@Gel in promoting macrophage polarization, Western blot analysis was performed. As shown in Figures 6G, H, Cur-Exo@Gel upregulates IκB-α expression, leading to a significant reduction in phosphorylated p65 levels, thereby decreasing the proportion of M1 macrophages and fostering an anti-inflammatory microenvironment conducive to orthopedic wound healing.

### 3.6 *In vivo* wound healing study

**FIGURE 7:**
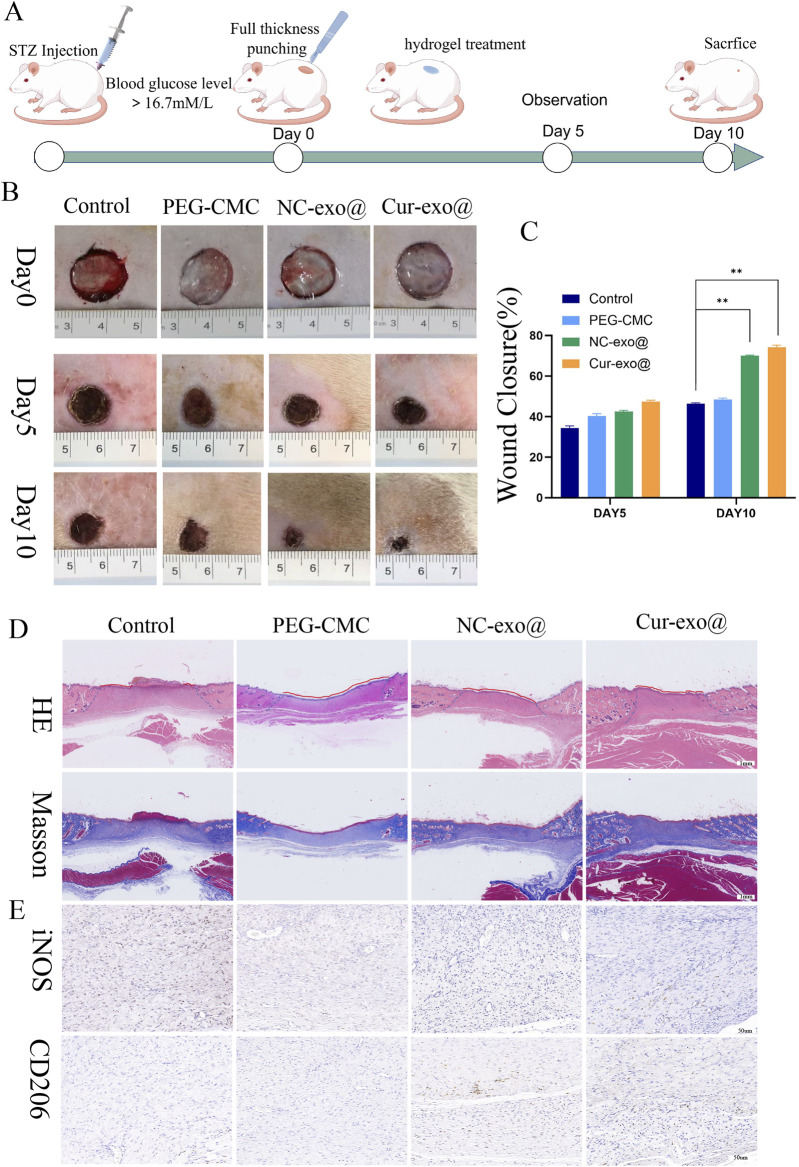
Wound healing with different hydrogels *in vivo*. **(A)** Preparation and observation of diabetic chronic wounds related to orthopedics. **(B)** Photographs of the wound treated with the hydrogel in different hydrogel groups. **(C)** Wound recovery curve of different hydrogel groups. **(D)** H&E staining and Masson staining of wound tissue on day 10 in different hydrogel groups. **(E)** IHC staining of wound tissue in iNOS and CD206.

To investigate the therapeutic potential of hydrogels for chronic orthopedic wound healing, a full-thickness skin defect model was established in STZ-induced diabetic rats (60 mg/kg, intraperitoneal injection) following acclimatization, as outlined in Figure 7A. A 15-mm diameter full-thickness skin defect was created on the dorsal surface of diabetic rats, followed by treatment with various hydrogels. Wound healing was assessed on days 5 and 10 post-surgery, with tissue harvesting performed on day 10. As shown in Figures 7B, C, the Cur-Exo@Gel group exhibited significantly reduced wound areas compared to the control group (*p* < 0.05). The NC-Exo@Gel group showed a comparable trend in wound closure, though the wound area remained slightly larger than that of the Cur-Exo@Gel group. These results demonstrate that both Cur-Exo@Gel and NC-Exo@Gel accelerate chronic wound healing, with Cur-Exo@Gel exhibiting superior efficacy. To further elucidate the therapeutic effects, H&E and Masson’s trichrome staining were conducted (Figure 7D), revealing that Cur-Exo@Gel significantly enhanced epithelial cell migration, reduced wound distance, and promoted collagen deposition. To explore the impact of inflammation regulation on macrophage polarization, IHC analysis of iNOS and CD206 was performed. The Cur-Exo@Gel group displayed a significant reduction in iNOS expression, which is indicative of decreased M1 macrophage activity, while CD206 expression showed no significant increase, suggesting limited enhancement of M2 polarization *in vivo* (Figure 7D).

## 4 Conclusion

In this study, we successfully developed a pH-responsive, highly biocompatible, injectable, and self-healing hydrogel composed of Cur-Exo and PEG-CMC, formed via Schiff base cross-linking. Our findings demonstrate that the hydrogel enables targeted exosome release in the weakly acidic microenvironment (pH 4.5) of diabetic orthopedic wounds, exhibiting excellent injectability and self-healing properties to meet the demands of irregular orthopedic wounds. *In vitro* experiments confirmed that Cur-Exo@Gel significantly mitigates endothelial cell oxidative stress, promotes endothelial cell migration, and enhances angiogenesis. Furthermore, Cur-Exo@Gel reduces the proportion of pro-inflammatory M1 macrophages by upregulating IκBα expression and suppressing p65 phosphorylation, thereby ameliorating the inflammatory wound microenvironment. *In vivo* studies using a diabetic rat full-thickness skin defect model revealed that Cur-Exo@Gel significantly reduced wound areas, with enhanced epithelial migration and collagen deposition. Immunohistochemical analysis further confirmed reduced iNOS expression, indicating suppressed M1 activity. This cell-free therapeutic strategy provides novel insights into the repair of chronic orthopedic wounds and offers a promising approach for clinical translation in regenerative orthopedics.

## Data Availability

The original contributions presented in the study are included in the article/supplementary material; further inquiries can be directed to the corresponding authors.
